# Development of an Easy-to-Operate Underwater Raman System for Deep-Sea Cold Seep and Hydrothermal Vent In Situ Detection

**DOI:** 10.3390/s21155090

**Published:** 2021-07-27

**Authors:** Qingsheng Liu, Jinjia Guo, Wangquan Ye, Kai Cheng, Fujun Qi, Ronger Zheng, Zhilei Sun, Xilin Zhang

**Affiliations:** 1 College of Information Science and Engineering, Ocean University of China, Qingdao 266100, China; liuqingsheng@stu.ouc.edu.cn (Q.L.); yewangquan@ouc.edu.cn (W.Y.); chengkai@ouc.edu.cn (K.C.); fujunqi@ouc.edu.cn (F.Q.); rzheng@ouc.edu.cn (R.Z.); 2 Qingdao Institute of Marine Geology, China Geological Survey, Qingdao 266071, China; zhileisun@yeah.net (Z.S.); ouczhxl@163.com (X.Z.)

**Keywords:** Raman system, in situ detection, deep sea, hydrothermal vent, cold seep

## Abstract

As a powerful in situ detection technique, Raman spectroscopy is becoming a popular underwater investigation method, especially in deep-sea research. In this paper, an easy-to-operate underwater Raman system with a compact design and competitive sensitivity is introduced. All the components, including the optical module and the electronic module, were packaged in an L362 × Φ172 mm titanium capsule with a weight of 20 kg in the air (about 12 kg in water). By optimising the laser coupling mode and focusing lens parameters, a competitive sensitivity was achieved with the detection limit of SO_4_^2−^ being 0.7 mmol/L. The first sea trial was carried out with the aid of a 3000 m grade remotely operated vehicle (ROV) “FCV3000” in October 2018. Over 20,000 spectra were captured from the targets interested, including methane hydrate, clamshell in the area of cold seep, and bacterial mats around a hydrothermal vent, with a maximum depth of 1038 m. A Raman peak at 2592 cm^−1^ was found in the methane hydrate spectra, which revealed the presence of hydrogen sulfide in the seeping gas. In addition, we also found sulfur in the bacterial mats, confirming the involvement of micro-organisms in the sulfur cycle in the hydrothermal field. It is expected that the system can be developed as a universal deep-sea survey and detection equipment in the near future.

## 1. Introduction

In recent years, the attempt of applying Raman spectroscopy to marine geochemical detection has achieved satisfactory results. Since the first deep ocean Raman in situ spectrometer system (DORISS) was reported in 2004 [1], researchers have established several underwater Raman systems and applied them to different in situ detection scenarios. The typical instruments include the upgraded DORISS II developed by Monterey Bay Aquarium Research Institute (MBARI) [2], the hybrid Raman insertion probe (Rip) developed by the Institute of Oceanology Chinese Academy of Sciences (IOCAS) [3], and the underwater Raman systems independently developed by the Ocean University of China (OUC) and the French Research Institute for Exploitation of the Sea (IFREMER) [4,5,6,7,8]. These systems have been deployed on ROVs for in situ detection of sediment pore water [9,10], deep-sea minerals [11,12], bacterial mats [13], methane hydrates [14,15,16], and the fluid of hydrothermal vents or cold seeps [17,18].

In these reports, deep-sea Raman systems were always treated as specific instruments rather than general-purpose sensors for their significant size and unique deployed mode. Most of the early underwater Raman systems adopted large scientific-grade spectrometers as the detection component to ensure sensitivity, which resulted in large system sizes and power consumption [19]. In addition, some systems were constructed with two cabins (a detection capsule and an optical probe), which makes their deployment and operation quite different from traditional sensors [2,3]. Usually, they require special modifications to adapt to the ROV system, such as modifying the mountings, changing the operation mode, and adjusting the ROV counterweights. These changes are not easy to implement, especially for equipment that takes up a large payload. Researchers have to balance the installation sequence and position of various sensors. On the other hand, some battery-driven underwater vehicles, such as autonomous underwater vehicles (AUVs), human-occupied vehicles (HOVs), and autonomous remotely operated vehicles (ARVs), are sensitive to the weight and the size of sensors. It is difficult for existing underwater Raman systems to meet the needs of these lightweight platforms. Therefore, a lightweight underwater Raman system with a low power cost was necessary. Employing a portable spectrometer instead of the large scientific-grade spectrometer seems to be a feasible solution. However, it could lead to another fatal problem, i.e., performance degradation. Despite portable spectrometers undergoing rapid development and being applied in many detection fields [20,21,22], they still have some performance gaps compared to large scientific-grade equipment. Merely replacing large components with a portable instrument will inevitably lead to performance degradation. Therefore, miniaturised underwater Raman systems require a more efficient optical layout. Meanwhile, except for the solved matters in the water, many solid targets of interest at seafloor hydrothermal and cold seep sites, such as elemental sulfur, carbonates, sulfates, sulfides, and organics, are Raman active. Hence, the optical system must consider different detection needs for both water and solid targets to meet the demands of investigating different targets in the deep sea.

This paper presents our attempt at constructing an easy-to-operate Raman system for deep-sea surveying and shows preliminary detection results obtained by this system in ocean survey applications.

## 2. System Configuration

### 2.1. Overview of the System

**Figure 1:**
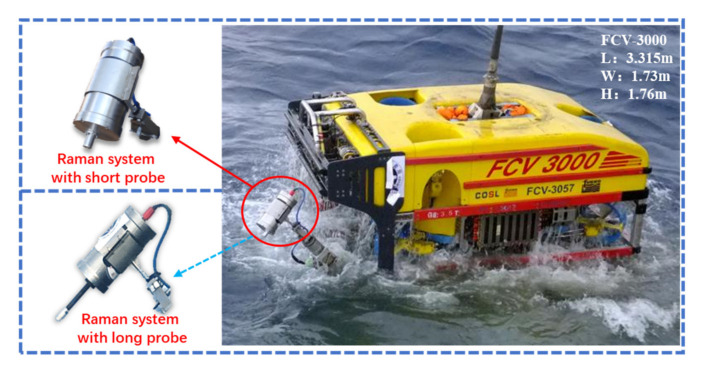
The system deployed on the 3000m class ROV for deep-sea investigation.

Figure 1 shows the newly developed Raman system deployed on the ROV "FCV3000" for deep-sea investigation. The system takes a compact single-capsule structure, and all the components are packaged in a titanium pressure vessel (L362 × Φ172 mm in size). The capsule contains an 11 mm thick titanium cylinder and two 31 mm thick caps, and it can withstand 50 MPa water pressure, with a safety margin of 25%. The total weight of the system is 20 kg in air and about 12 kg in water. Hence, it can be easily operated by the hydraulic manipulator of the ROV.

This system has two interchangeable optical probes, as shown in Figure 1: a short universal probe and a long insert probe. The short probe is compact and easy to deploy, which can be used as a conventional system configuration installation on the front cap. The long optic takes a high-temperature-resistant design. It can be directly inserted into the hydrothermal vents to detect the internal fluid. It can also afford to detect targets in confined spaces, such as rock crevices. These two probes have the same interface parameters and can be interchanged according to the mission style. On the end cap, a 13-pin waterproof connector(DBH13F, SubConn Inc., Burwell, NE, USA) was installed as the electrical interface. The Raman system can connect with the ROV to obtain power and communicate with the deck terminal via this connector. With the powerful communication capabilities of the ROV based on its umbilical cable, the system can operate in either interactive mode or automatic mode.

Due to its small size and lightweight design, the system has a flexible deployment model. On the one hand, it can be directly fastened to the ROV manipulator and execute detection by moving the manipulator. On the other hand, it can also be placed in the sampling basket and operated by the manipulator when needed. The "FCV3000" is a 3000-meter class engineering ROV, which equips a five-function manipulator (Schilling Rig Master, TechnipFMC, UK) and a seven-function manipulator (Schilling Titan 4, TechnipFMC, UK), but without a professional sampling basket. Therefore, we adopted the first deployment method of fixing the system on the five-function manipulator, as shown in Figure 1, and we left the seven-function manipulator for other operations, such as sediment or seawater sampling. The detailed specifications of the easy-to-operate Raman system are shown in Table 1.

It is always a challenge to maintain detection performance while miniaturising equipment. The performance gap between portable spectrometers and large scientific-grade ones is evident. Therefore, enhancing signal collection capabilities and reducing losses in the detection seems the only viable way to reduce this gap. The newly developed system employs optimised focusing lens parameters to accommodate the solids detection without sacrificing the sensitivity of detecting solutes in water. Meanwhile, the low-loss optical structure helps the system achieve a comparable detection capability to existing underwater Raman systems, even better than some huge equipment. In addition, the compact, single capsule design dramatically simplifies the deployment and operation of the system. In practice, the compact system brings a considerable improvement in the user experience. In Table 2, some critical features of the developed system are listed and compared with some reported underwater Raman systems. The progress in miniaturisation and sensitivity of the easy-to-operate system is evident.

### 2.2. Optical Layout

**Figure 2:**
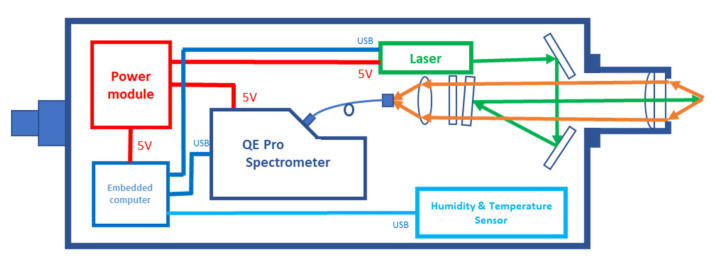
Schematic diagram of the easy-to-operate Raman system.

Figure 2 shows a schematic diagram of the newly developed system. It consists of a waterproof capsule, a laser, a spectrometer, the necessary excitation/collection optics, and some electronic modules. Most of the components are commercially available, except for the excitation/collection optics. Adopting proved commercial components will contribute to reducing construction costs and increasing the stability of the system. Although the integrated excitation/collection optics can also be obtained from optical manufacturers, they usually lack specific optimisation for underwater detection applications, such as working distance and system aperture. These parameters are closely related to the system detection ability and should be carefully treated. Therefore, we built a particular excitation/collection optics module for the underwater Raman spectroscopy system. In this section, we presented our unique optical layout design.

#### 2.2.1. Spectral Coverage

In the hydrothermal area and cold seep environments, various chemical, physical, and biological processes are integrated and influence each other to form a complex ecosystem based on chemosynthetic symbioses. In this ecosystem, microbiology plays a connection role in energy translation and element recycles. The potential detection targets in the hydrothermal vents and cold seeps include various minerals (such as carbonates, sulfates, and sulfides), gases (such as oxygen, nitrogen, carbon dioxide, hydrogen sulfide, methane, and ethane), dissolved matter (such as carbonate ions and sulfate ions), gas hydrates, and some organic compounds. These targets are typically widely distributed. The characteristic peaks of sulfur and sulfur-containing minerals are mainly located in the low-wavenumber range around 200–500 cm^−1^. For most minerals and gases, such as CO_2_, O_2_, and N_2_, the Raman peaks are mainly located mid-range around 500–2000 cm^−1^. For organic compounds and the O–H groups of clathrate hydrates and hydroxylated minerals, such as zeolites and clays, the peaks are mainly located in the high range around 2000–4000 cm^−1^. The Raman peaks of some typical targets are shown in Table 3.

To satisfy the requirement of detecting cold springs and hydrothermal zones, the underwater Raman spectroscopy system should have broad spectral detection capability, considerable sensitivity, and appropriate spectral resolution. In addition, it is desirable that the system can accommodate the detection of fluorescent targets that do not have Raman peaks but are important for researching the marine environment, such as chlorophyll et al. This system offers a spectral range from 200 cm^−1^ to 4500 cm^−1^ and an approximately 5 cm^−1^ resolution to meet different underwater target detection needs. Compared with the vast expert Raman spectrometer, the newly developed system has disadvantages in detection sensitivity and spectral resolution. By using deconvolution and peak fitting, the problem of low resolution can be alleviated to some extent. However, the problem of low detection sensitivity must be improved by system optimisation.

#### 2.2.2. Excitation Source

According to the Raman scattering mechanism, both the excitation power and the wavelength can affect the Raman scattering signal intensity. It is a relatively simple method to enhance the signal intensity by increasing the laser energy. However, this method has some limitations in practical application. Increasing the excitation laser power can effectively improve the system detection ability for targets with a high damage threshold, such as water and gas. However, the high-energy laser may damage the samples for targets with a low damage threshold, such as biological samples and dark minerals. Moreover, a high-energy laser implies more significant power consumption and space occupied, which is not conducive to system miniaturisation. Therefore, the more feasible method is using an energy-tunable laser with appropriate power while reducing the coupling loss of the excitation laser to improve efficiency.

Conventional underwater Raman systems usually adopt a multi-capsule structure to balance the contradiction between the enormous size and the requirement of easy-to-operate. In such a structure, the laser and excitation/collection optics are encapsulated in separate chambers and connected by a fibre optic. So that laser beam needs two reshape processes: coupling into the fibre and output collimation. These processes always exhibit laser-coupling loss, completely determined by the degree to which the optics depart from ideal due to the optical system’s aberrations and misalignments [23]. Therefore, we use a single-cabin structure to encapsulate all the components in the same cabin and employ mirrors to guide the laser beam to avoid fibre-coupling loss. By comparing the energy output from the laser with the measured value by the energy meter at the probe tip, we can estimate the overall laser loss in the system. Actual results show that by adopting this method, the loss rate of the laser in the system is reduced to 9%.

The wavelength of the laser is another critical factor that can affect detection efficiency. The typical excitation source used for underwater Raman spectroscopy is a 532 nm laser or 785 nm laser. The selection of the excitation wavelength requires a comprehensive consideration of various factors. First, the Raman scattering intensity follows the same fourth-power law as Rayleigh scattering, whereby the Stokes and anti-Stokes Raman band intensity is proportional to (λ_0_ − λ)^−4^ [24]. This means that shorter wavelengths lead to better excitation efficiency. The second factor that should be considered is detector quantum efficiency. Most silica-based detectors have better response performance in the visible range, which drops off sharply as they approach the infrared or ultraviolet range. As shown in Table 3, for most targets, with 532 nm as the excitation source, almost all peaks are in the visible band. Hence, 532 nm is more advantageous in terms of detector response. However, this poses another problem: fluorescence interference. The Raman peaks located in the visible band always suffer from fluorescence interference. However, taking the 785 nm laser can ideally avoid this problem because both the laser and signal are far from the fluorescence band. Furthermore, water absorption is also a crucial factor in wavelength selection. Coincidentally, seawater has good transmission efficiency in the visible-light band, especially blue and green bands. Unlike the rapid decay of the 785 nm light, the 532nm laser can adapt to longer working distance detection tasks in water. According to the previous research [11], significant fluorescence was not observed for the hydrothermal and cold seep minerals analysed using green excitation. In addition, with appropriate baseline calibration correction algorithms, broad fluorescence peaks can be removed without causing unacceptable losses to the Raman signal. Hence, the system employs a 532 nm narrow-line laser (08-DPL, Cobolt, Stockholm, Sweden) as the Raman excitation source. The laser is compact and provides variable output power from 0 to 200 mw, covering the detection needs of materials with high or low damage thresholds.

#### 2.2.3. Excitation/Collection Optical Layout

**Figure 3:**
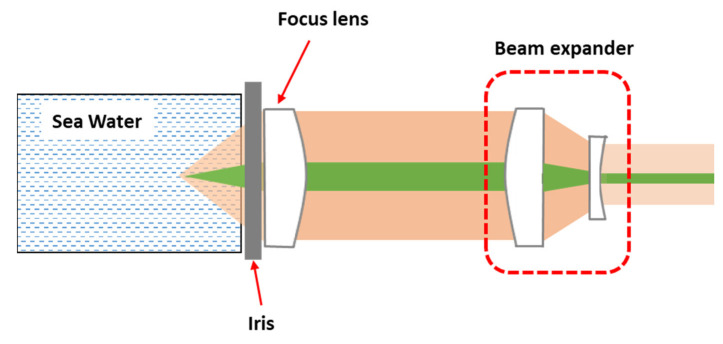
Schematic diagram of the tunable-aperture setup.

The excitation/collection optics system includes the necessary mirrors, lenses, filters, and essential adjustment mounts. As shown in Figure 2, after being emitted from the laser, the beam is reflected by two high-reflectivity mirrors (BB05-E02, Thorlabs, US) and irradiated on a long-pass filter (LP03-532RU-25, Semrock, US) at a small angle. Here, the long-pass filter is used for laser reflection and the attenuation of Rayleigh scattering light by rotating at a small angle (<5°). Then, the laser beam passes through the focusing lens and, finally, focuses on the detection target. In contrast, the stimulated Raman signal is collected by the focusing lens and transmitted backwards. After filtering the Rayleigh scattering, the Raman signal is coupled into the optical fibre and, finally, guided to the spectrometer for detection. When the internal optical components do not restrict the system’s aperture, the signal strength only depends on the signal collection ability of the focusing lens. To evaluate this ability, we introduce f-number(F/#)–a parameter usually used to illustrate the light-gathering properties. F/# is defined as follows:

(1) $$F/\#=\frac{f}{D}$$

where *f* represents the focal length, and *D* is the aperture of the lens. A smaller F/# denotes that the focusing lens has better signal collection capability. According to Equation (1), either increasing the aperture or decreasing the focal length can reduce F/#. However, it is difficult for marine equipment to decrease F/# by increasing the optical window’s aperture. An increase in optical aperture necessitates a dramatic increase in window thickness, which brings challenges related to the seal and increases the system volume. Meanwhile, a sizeable optical window is easily scratched, bringing potential danger to the system. Therefore, a feasible method involves changing the focal length to improve the system’s collection efficiency. For water and gases, the focal length of the focusing lens can be very small because they are transparent, and the laser’s focus point always can fall into the inside of them. An extreme approach is to use a spherical lens to focus the laser and collect the signal. However, things become complicated when it comes to solid targets, such as minerals, bacterial mats and shells. These samples often have rough surfaces; thus, focusing must be considered for their detection. On the other hand, the focusing lens also needs a suitable working distance to enable nonintrusive detection and avoid collisions between the window and the target. Furthermore, a considerable depth of focus is beneficial for solid sample detection; it can increase the system tolerance toward the focus position, which is crucial for systems without a precise positioner. However, the depth of focus is approximately proportional to the square of F/# [25], as shown below:

(2) $$L\propto \lambda {\left(\frac{f}{D}\right)}^{2}$$

where L is the depth of focus, and λ is the laser wavelength. The requirement of solid detection must be balanced with water detection. To obtain the specific parameters, we set up a tunable-aperture experimental system based on the introduced optical structure mentioned above to explore the relationship between signal strength and F/#. The specific experimental setup is shown in Figure 3.

**Figure 4:**
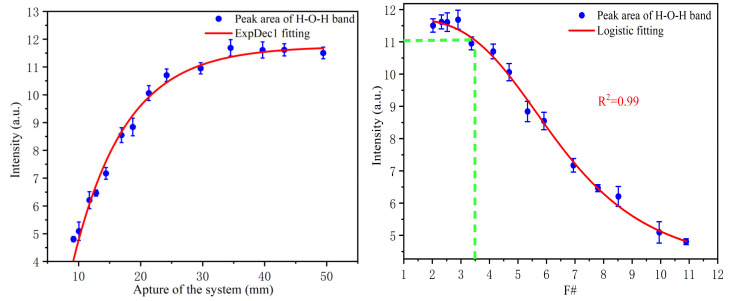
The experiment results showing the collected Raman signal intensity of water as a function of the aperture (**left**) and F/# (**right**) of the system.

In this system, we used a beam expander to enlarge the system aperture from 25.4 mm to 50.8 mm to increase the sampling point density and reduce the measurement error. At the same time, a tunable iris was used to change the system aperture and keep the focal length constant (100 mm). This tunable iris can continually change the aperture from 9 mm to 50.8 mm so that the experiment system can operate in the F/# range from 2 to 11. We chose the peak area of the H–O–H bending band (1640 cm^−1^) as the index to measure the spectral intensity because this peak is not sensitive to external factors and is often used as an internal standard for the quantitative analysis of solute in water. Furthermore, we converted the aperture value to F/# according to Equation (1). The result is shown in Figure 4.

The experimental results show that the relationship between the system aperture and signal strength is nonlinear. At the initial stage, the signal strength increases rapidly with the change in system aperture. After reaching a particular strength, the growth rate begins to slow. This trend is determined by both the Raman scattering function and the lens collection property. It can be seen from the experimental results that when the aperture is about 30 mm, the signal strength is nearly stable. After converting to F/#, this conclusion can be extended to the focusing lens system with arbitrary focal length. The maximum F/# can be extended to about 3.5 without obviously affecting the water signal intensity. Although this is an experimental conclusion, it also reflects the relationship between the system aperture and the signal strength to some extent. In the probe design, we followed this rule to select the focus lens.

The deep-sea Raman system fixed a focusing lens with a cap-edge-step shape into a pressure-tolerant metal tube as the detection probe. As shown in Table 4, two types of probes are equipped for the system: short universal optics and long insert optics. The short sampling probe is 60 mm long and can provide a 25 mm working distance in water with a 10 mm aperture. Due to its advantages in deployment, the short probe was installed on the system to detect seawater and solid targets. The long insert probe is 178 mm long and can supply an 18 mm working distance with a 6 mm aperture. It can be used to detect target objects in a narrow space, such as rock crevices. To better accommodate operations in confined spaces, we slightly relaxed the F/# limit for long probes to obtain better-focusing robustness. By extending the F/# from 3.5 to 3.8, the focusing depth of the long probe is doubled with only a minor signal loss. After installing the metal filter, the long probe can be inserted directly into the hydrothermal vent to detect hydrothermal fluid. In the long insert probe, FFKM material O rings were used for sealing. This material’s theoretical working temperature can reach 327 °C, and it is feasible to stand the 300°C in a practical working environment.

### 2.3. Electronic Module

The electronic module contains a power converter, a temperature & humidity sensor, and an embedded computer. Specifically, the power converter is responsible for converting the power from the ROV system to a general 5 V DC power and supplying it to the internal electrical equipment. The temperature & humidity sensor monitors the cabin environmental parameters and alerts when the system overheats or seawater leaking. The embedded computer (PCM-3363, Advantech, Taibei, Taiwan) acts as the control and communication centre. It can operate in two working modes: interactive mode and automatic mode. In interactive mode, the embedded computer and the deck server jointly constitute a master-slave control network. As the slave machine, the embedded computer synchronises the collection parameters from the deck server and controls the equipment to carry out the spectrum collection. Then, the collected data will be transmitted back to the deck server for display in real-time meanwhile backing up locally. The deck server is only responsible for setting the acquisition parameters and displaying the results uploaded by the embedded computer at the terminal. This method requires a stable real-time communication channel, including a network or serial communication channel. Conversely, the embedded computer works independently in automatic mode to perform spectral acquisition with preset parameters and save the results on the internal data storage unit. This mode is suitable for underwater platforms with poor communication or lacking effective communication channels.

## 3. Results and Discussion

### 3.1. System Evaluation in Terms of the Limit of Detection and Stability

Sulfate ions are one of the major macronutrient ions in seawater, whose content changes usually accompany significant physical or chemical processes. For example, in marine sediments, sulfate ions can be converted to sulfur ions by the combined action of sulfate-reducing and methane-oxidising bacteria and form insoluble minerals with elements such as cobalt and cadmium in the environment. This results in a dramatic reduction of sulfate ions in the pore water, especially for the cold seep and hydrothermal vent areas, which are rich in methane or hydrogen sulfide. Meanwhile, sulfate ions are the only component that can be detected by the deep-sea Raman system in ordinary seawater. So, it is usually used as the standard sample to evaluate the detection capability in laboratory tests. In this paper, we prepared five gradient solutions (0.5 mmol/L, 1.0 mmol/L, 5.0 mmol/L, 10.0 mmol/L, 15.0 mmol/L) to test the detection limit and linearity of the system.

**Figure 5:**
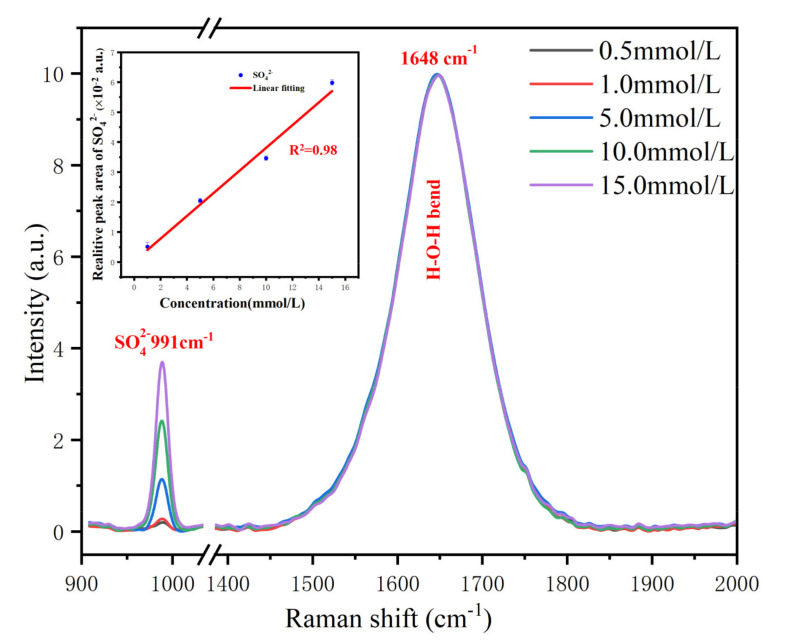
Raman spectra of Na_2_SO_4_ solution with different concentrations (0.5–15.0 mmol/L) taken with 8 s integration. The insert shows the linear fitting result.

In the tests, the detector exposure time was set as 8 s, and each sample took 10 spectra on average. The test results are shown in Figure 5. To determine the system LOD, we took the 3σ principle as the evaluation criterion in this paper. The 3σ principle is one of the normative approaches for establishing the limit of detection (LOD) defined by the International Union for Pure and Applied Chemistry. It uses the average blank signal value as the reference point and three times of standard deviations of the blank as LOD. In test results, the signal to noise ratio (SNR) for the 1.0 mmol/L solution was 5, better than the 3σ defined LOD criterion. However, for the 0.5 mmol/L solutions, the SNR was only about 2. Therefore, the LOD is a concentration between 0.5 mmol/L and 1.0 mmol/L. After further calculation with the 3σ principle, the LOD was determined as 0.7 mmol/L.

Water is the most dominant part of seawater, occupying about 96.5% of the total mass, and changes in solute concentration have a negligible effect on it. Therefore, it is usually used as a constant internal standard for the quantitative analysis of solutes. Furthermore, taking the peak of the water H–O–H bending band (1640 cm^−1^) as a constant reference, the sulfate peak can be normalised and used for quantitative analysis. The normalised concentration gradient results showed an excellent linear property, and the fitting parameter *R^2^* can reach 0.98 over the whole range.

**Figure 6:**
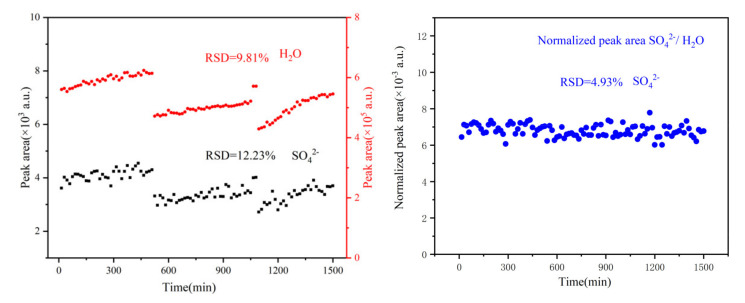
Results of the 24h torture test. Peak area of water and sulfate (**left**); normalised peak area of sulfate using water as an internal standard (**right**).

In particular, to assess the system’s long-term stability, a 24 h torture test was carried out with seawater, and the results are shown in Figure 6. As presented on the left of Figure 6, both the water and the sulfate signals showed apparent fluctuations. This fluctuation can be divided into two parts: (1) a significant fluctuation with the same trend for both water and sulfate; (2) a slight and random vibration. The former was mainly caused by laser energy, whereas the latter was primarily dominated by noise.

The Raman signal of water was two orders stronger than that of sulfate. Therefore, the peak area of sulfate was more sensitive to variations in noise. Reflecting on the results, the relative standard deviation (RSD) of sulfate will be higher than water. Indeed, as shown in Figure 6, the RSD of the water peak area was 9.81% better than that of sulfate at 12.23%. After normalising the sulfate peak area using the water peak area as an internal standard, the RSD of the sulfate peak area decreased to 4.93%, as shown on the right of Figure 6. This means that the system can still guarantee measurement fluctuations below 5% over long periods. Altogether, the results demonstrate that the system’s detection ability is suitable for the deep sea investigation.

### 3.2. Sea Trials and Results

In October 2018, the developed Raman system carried out its first sea trial by deploying on the 3000 m class ROV "FCV3000". During the cruise, over 20,000 spectra were captured from excitation targets, and the maximum dive depth of the system was 1038 m. Some of the typical target results were described below.

**Figure 7:**
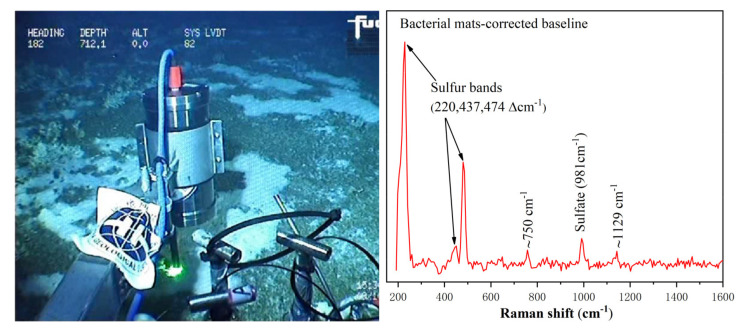
The image taken by the ROV camera showing the easy-to-operate system was in operation (**left**), and the resultant spectrum collected from the bacterial mat (**right**).

During the hydrothermal region cruise, a large number of white bacterial mats were found around the hydrothermal vent. We operated the manipulator to bring the Raman system close to the mats for detection. The field graph took by the ROV camera and the spectrum of bacterial mat obtained in the hydrothermal field are shown in Figure 7. The raw data were baseline-corrected using the asymmetric least square method to remove the fluorescence background. It can be seen that there were clear Raman peaks at 220, 437, 474, 750, 981, and 1129 cm^−1^. The Raman peaks at 220, 437, and 474 cm^−1^ were attributed to the primary bands of S_8_. The 981 cm^−1^ Raman signal represented sulfate in seawater, and the peak at 1129 cm^−1^ revealed the presence of β-carotene. Previous studies have shown that large bacteria morphologically affiliated with the genera *Beggiatoa* and *Thiothrix* are the dominant morphotypes in these white bacterial mats [26,27,28]. These organisms, members of the order *Thiotrichales* (*Gammaproteobacteria*), are known for their ability to form biofilms on oxic/anoxic interfaces, using dissolved free oxygen to oxidise reduced sulfur compounds [29]. Indeed, the discovery of S_8_ in the Raman spectrum of the bacterial mats also confirms the role of these bacteria in the deep-sea sulphur cycle.

**Figure 8:**
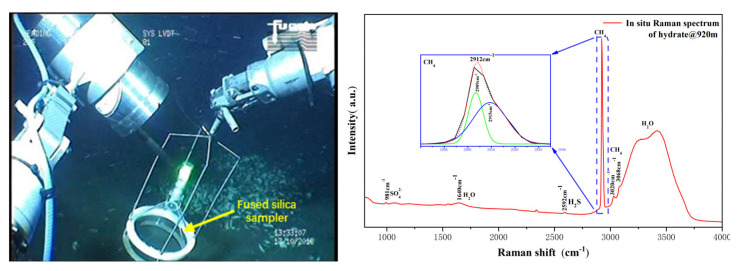
The image showing the easy-to-operate system is working with a fused silica sampler (**left**) and a typical spectrum obtained from the in situ formed gas hydrate (**right**).

In the cold seep investigation, we used a fused silica sampler to collect the seepage gas and detected it using the developed Raman system. The field graph and the resultant spectrum of the gas hydrate formation experiment are shown in Figure 8. In this spectrum, in addition to the sulfate Raman peak at 981 cm^−1^ and water molecule Raman peaks at 1640 and 3395 cm^−1^, several peaks assigned to hydrocarbon molecules were revealed. The strong Raman peak of methane at 2912 cm^−1^ and two much weaker bands at 3020 cm^−1^ and 3068 cm^−1^ were observed in the spectrum. There was also a weak band located at 2592 cm^−1^, which was assigned to H_2_S. The peak at 2912 cm^−1^ was caused by the C–H stretching of methane hydrate, and it contained two isolated peaks, indicating the methane molecule occupied large and small cages, respectively [18,30]. Meanwhile, the Raman peaks at 3020 cm^−1^ and 3068 cm^−1^ revealed the existence of free methane gas [14]. From the above information, we can infer that the synthetic hydrate was still in the growth phase, while free gas was measured along with the hydrate phase.

**Figure 9:**
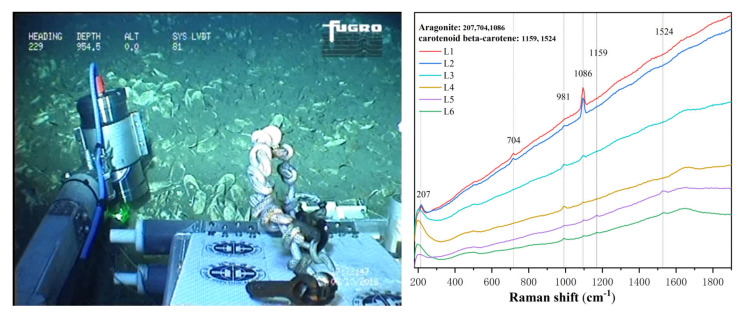
The image showing the easy-to-operate system is detecting the clamshell in the abandoned cold seep field (**left**) and the obtained spectra from the clamshell taken in different locations (**right**).

Figure 9 presents the field graph and the spectra collected from clamshells in the cold spring area. This location was a large abandoned clam field that used to be an active cold seep; many clamshells scattered on the seabed covering hundreds of square meters. By adjusting the position of the manipulator, the system collected six valuable spectra (L_1_–L_6_). In the Raman spectra L_1_ and L_2_, we can see the typical aragonite characteristic peaks located at 706 and 1086 cm^−1^ contributed by the aragonitic phase carbonates in the shell. Some other spectra, such as L_5_ and L_6_, also showed weak carotenoid characteristic peaks at 1159 and 1542 cm^−1^. However, the characteristic peaks of carotenoids were not consistent with the intensity changes of aragonite characteristic peaks, indicating that carotenoids did not come from shells but were more likely caused by bacterial mats covering the shell or sediment surface. In the spectra of L_3_ and L_4_, all peaks, except for the sulfur peak at 981 cm^−1^, are very weak. This phenomenon occurred because the laser beam was not accurately focused on the sample surface; thus, the focus point fell into the water.

## 4. Conclusions

The newly developed underwater Raman system demonstrates significant advantages in flexibility and convenience by taking a more compact single capsule design. It significantly reduces the threshold of using underwater Raman systems and facilitates its application on lightweight underwater platforms. The laboratory tests showed that with the low-loss optical layout, the detection sensitivity of the system was close to the existing underwater Raman system. Furthermore, torture tests also proved the system stability in the long term application. The developed system has been successfully applied in the deep sea survey and completed the in situ detection of typical targets in hydrothermal vent and cold seep. After sufficient testing, the device is expected to be equipped on ROVs as a universal device.

## Figures and Tables

**Table 1:** Specification of the easy-to-operate Raman system.

Module	Apparatus	Specifications
Mechanics	Chamber	Material: TC4
		Size: 362 × 172 mm (length × diameter)
		Weight: 20 kg (in air)/12 kg (in water)
		Maximum work depth: 5000 m
Optics	Exciting wavelength	Wavelength: 532 nm
		Energy: 0–200 mW
	Spectral range and resolution	Spectral range: 537–700 nm
		Spectral resolution: ~0.16 nm
	Short universal probe	Length: 60 mm long
		Aperture: 10 mm
		Work distance (from the target to the optic window): 25 mm
		Pressure resistance: 50 MPa
	Long insert probe	Length: 178 mm
		Aperture: 6 mm
		Work distance (from the target to the optic window): 18 mm
		Pressure resistance: 50 MPa
	Layout	Backscattering collection
Electronics	Power supply	24V DC from ROV (≥60 W)
	Embedded computer	Advantech PCM-3363
	Communication	Ethernet (10–100 Mbps)
		Serial port (RS-232)

**Table 2:** The parameters of the developed system in comparison with the reported underwater Raman system.

Property	DORISS (MBARI)	DORISSII(MBARI)	DOCARS (OUC)	RiPs (IOCAS)	The Developed System
Dimension	1450 × 720 × 420 mm (L × W × H)	305 × 762 mm (Φ × L)	258 × 800 mm (Φ × L)	/	172 × 362 mm (Φ × L)
Weights	220 kg in air/66 kg in water	140.6 kg in air	60 kg in air	/	20 kg in air/12 kg in water
Structure	Two capsules plus probe	One capsule plus probe	One capsule	One capsule plus probe	One capsule
Exciting wavelength	532 nm	532 nm	532 nm	532 nm	532 nm
LOD(SO_4_^2−^)	1.6 mmol/L	\	0.4 mmol/L	0.6 mmol/L	0.7 mmol/L
Working depth	≤4000 m	≤4000 m	≤4000 m	≤6000 m	≤4000 m

**Table 3:** The Raman shift and peak location of the typical targets in deep sea research.

	Sample (Composition)	Mode	Raman Shift (cm^−1^)	Peak Location * (nm)
Mineral	Anhydrite (CaSO_4_)	ν_1_	1017	562.4
		ν_2_	417, 499	544, 546.5
		ν_3_	1110, 1128, 1160	565.4, 565.9, 567
		ν_4_	608, 628, 675	549.8, 550.4, 551.8
	Gypsum (CaSO_4_·2H_2_O)	ν_1_	1008	562.1
		ν_2_	415, 494	544, 546.4
		ν_3_	1136	566.2
		ν_4_	620, 671	550.1, 551.7
	Barite (BaSO_4_)	ν_1_	988	561.5
		ν_2_	452, 462	545.1, 545.4
		ν_3_	1141	566.4
		ν_4_	617	550.1
	Pyrite (FeS_2_)	ν_1_	343	541.9
		ν_2_	379	542.9
		ν_3_	430	544.5
	Marcasite (FeS_2_)	ν_1_	323, 386	541.3, 543.2
		ν_2_	342, 377	542.9, 543.2
	S_8_	ν_1_	476	545.8
		ν_2_	219	538.3
		ν_3_	411	543.9
		ν_4_	234.4	538.7
	Calcite	ν_1_	1099	565.0
		ν_2_	876	558.0
		ν_3_	1435, 1444	576.0,576.3
		ν_4_	724	553.3
	Aragonite	ν_1_	1085	564.6
		ν_3_	1464, 1466	576.9, 577.0
		ν_4_	704	552.7
Gas	CO_2_	ν_1_	1387	574.4
		2ν_2_	1285	571.0
	CH_4_	ν_1_	2917	629.5
		ν_2_	1534	579.3
		ν_3_	3020	633.8
		ν_4_	1306	571.7
		2ν_2_	3068	635.8
	H_2_	Q_1_ (0)	4162	683.3
		Q_1_ (1)	4156	683.0
		Q_1_ (2)	4144	682.5
		Q_1_ (3)	4126	681.6
	H_2_S	ν_1_	2592	617.1
Solution	SO_4_^2−^	ν_1_	981	561.3
	CO_3_^2−^	ν_1_	1064	563.9
		ν_2_	880	558.1
		ν_3_	1395	574.6
		ν_4_	686	552.1
	HCO_3_^−^	ν_3_	1364	573.6
		ν_5_	1011	562.2
	HS^−^	ν_1_	2573	616.4
	H_2_O	ν_1_	3450	651.6
		ν_2_	1640	582.9
		ν_3_	3650	660.2
Organic	β-Carotenoid	ν_1_	1515	578.6
		ν_2_	1130	566.0
		ν_3_	1000	561.9
		ν_4_	1270	570.5

* Raman peak location with 532 nm excitation wavelength. The chlorophyll fluorescence peak at 675 nm has not been listed.

**Table 4:** Specifications of the changeable probes.

Probe	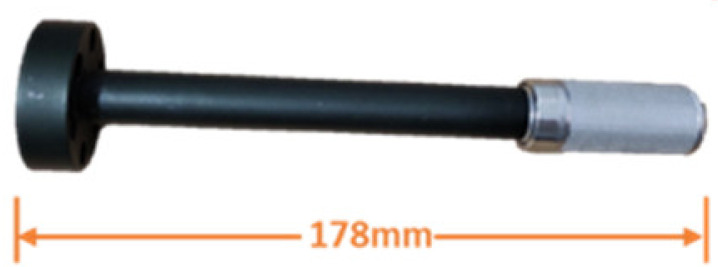	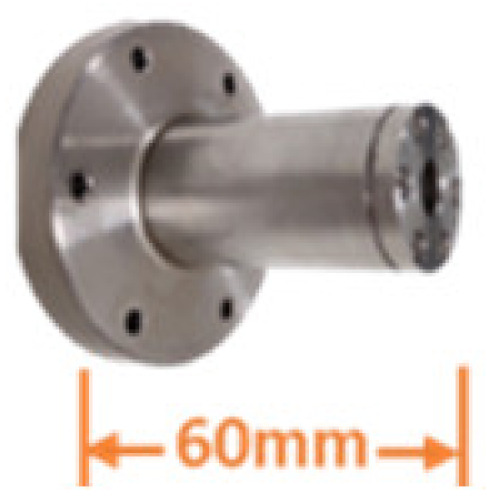
Focal length (in water)	23	35
Working distance in water	18	25
Aperture	6	10
F/#	3.8	3.5

## Data Availability

Data sharing is not applicable.
